# Comparing feedback learning and arousal responses in Down, Fragile X, and Williams syndromes

**DOI:** 10.1038/s41539-026-00438-2

**Published:** 2026-07-16

**Authors:** Astrid E. Z. Hallman, Charlotte Willfors, Matilda A. Frick, Ann Nordgren, Johan Lundin Kleberg

**Affiliations:** 1 Department of Psychology, Stockholm University, Stockholm, Sweden; 2 Department of Molecular Medicine and Surgery, Karolinska Institute, Stockholm, Sweden; 3 Department of Clinical Genetics and Genomics, Karolinska University Laboratory, Karolinska University Hospital, Stockholm, Sweden; 4 Child and Adolescent Psychiatry, Department of Medical Sciences, Uppsala University, Uppsala, Sweden; 5 Department of Laboratory Medicine, Institute of Biomedicine, University of Gothenburg, Gothenburg, Sweden; 6 Department of Clinical Genetics and Genomics, Sahlgrenska University Hospital, Region Västra Götaland, Gothenburg, Sweden; 7 Department of Clinical Neuroscience, Karolinska Institute, Stockholm, Sweden

**Keywords:** Neuroscience, Psychology, Psychology

## Abstract

Mechanisms underlying feedback learning in intellectual disability (ID) remain poorly understood. Down syndrome (DS), Fragile X syndrome (FXS), and Williams syndrome (WS) are genetic syndromes associated with ID, with distinct attention and arousal regulation profiles. Pupil dilation is a well-established index of feedback processing in typical development; little is known about these processes in non-social learning in these syndromes. Participants with DS (*n* = 13), FXS (*n* = 13), WS (*n* = 27), and typically developing (TD) individuals (*n* = 56) aged 6–60 years completed a reward contingency reversal task while eye-tracking recorded pupil dilation and gaze allocation. Data were analyzed using Bayesian mixed-effects models. All groups effectively learned from feedback, staying with rewarded options and switching after losses. WS participants performed similarly to TD participants. DS and FXS individuals showed more variable behavioral performance, indicating potential individual differences in feedback processing that warrant further investigation. Physiologically, TD individuals showed greater pupil dilation following losses than wins, whereas WS individuals showed no difference, suggesting attenuated physiological sensitivity to feedback valence. All groups tended to fixate on the previously rewarded options. This research highlights the value of combining behavioral and physiological approaches in ID research.

## Introduction

Intellectual disability (ID) is a condition characterized by intellectual impairment and challenges with adaptive behavior^1^, with heterogeneous etiology and symptoms. Although learning difficulties are a hallmark of ID, we know little about the cognitive and physiological processes underlying learning in this group. Learning to associate actions with rewards through feedback is known as *reinforcement learning* and is fundamental to daily life. Behaviorally, reinforcement learning results in an increased frequency of previously rewarded actions and a decrease in actions followed by omitted rewards or negative feedback^2^. So far, research regarding feedback learning processes in individuals with ID remains limited. This is surprising because commonly used interventions (e.g., applied behavior analysis) are built on principles from reinforcement learning^3^. Down syndrome (DS), Fragile X syndrome (FXS), and Williams syndrome (WS) are conditions associated with ID with a well-established genetic etiology. These genetic syndromes present unique challenges and characteristics not only in general intelligence but also in areas such as attention, executive function, and arousal regulation. Although these genetic syndromes are typically associated with ID, limited research exists on how learning occurs, particularly regarding the physiological processes involved. By studying the syndromes separately, we can better understand the variations within the ID population and the different needs of its various subgroups.

This study aims to examine the interplay between genetic etiology, learning behavior, and physiological arousal in ID, focusing on DS, FXS, and WS. Specifically, we examined task-evoked pupil dilation, a measure of arousal closely linked to the brain’s locus coeruleus-noradrenergic (LC-NE) system^4^, which is known to be sensitive to reward and feedback processing in typical^5,6^ and atypical development^7^. Recent research has explored the relationship between task-evoked pupil dilation and decision-making in conditions with altered reward processing, such as depression^8,9^ and individuals with high levels of eating disorder symptoms^10^. However, this relationship has not yet been studied in genetic syndromes associated with ID, like DS, FXS, and WS. Direct comparisons of feedback processes between different genetic conditions associated with ID can give novel insights into the heterogeneity of ID.

Across DS, FXS, and WS, the severity of ID ranges from mild to severe^11–13^. The degree of impairments varies both between and within syndromes; for example, female individuals with FXS typically present less severe cognitive difficulties than male individuals with FXS^12,14^. While all three syndromes are characterized by difficulties with attention and executive functions^11,15–18^, they differ in arousal regulation and reward sensitivity. WS is characterized by *hypoarousal*^19,20^, whereas FXS is characterized by *hyperarousal*^21,22^. In DS, findings suggest altered autonomic reactivity during attention tasks, with greater overall pupil dilation and reduced phasic responses to target stimuli compared to typically developed (TD) children^23^.

As mentioned, research on feedback processing in DS, FXS, and WS is limited. A recent study comprising a series of experiments on adapted versions of the Weather Prediction Task, a probabilistic learning task used to assess learning strategies, found that individuals with WS (age range 12–47 years) and DS (age range 9–32 years) could adapt their decisions following a deterministic reward schedule^24^. In contrast, they struggled when learning required interpreting probabilistic cue–outcome relationships, suggesting that deterministic feedback significantly facilitates task performance, whereas probabilistic feedback may hinder learning in this context^24^. In a computerized probabilistic learning task, it was shown that individuals with WS and individuals with ID of mixed etiology showed an increased sensitivity to losses compared to TD individuals^25^. Similarly, adult individuals with FXS also showed greater sensitivity to negative feedback in a probabilistic reversal learning task compared to TD individuals^26^. Taken together, these findings indicate heightened sensitivity to losses and challenges with learning from reward schedules that are not deterministic, yet little is known about the physiological mechanisms underlying feedback processing in these groups.

Making errors and receiving feedback after your actions drives learning. According to the reinforcement learning model, learning is driven by the difference between *expected* and *actual* outcomes, known as prediction error^27,28^. Feedback, therefore, signals both information about the value of the received reward and the need to update previous predictions (e.g., surprise or prediction errors). Recent research indicates that pupil dilation, which is closely linked to changes in LC-NE activity^4^, is sensitive to both surprise^8,29^ and reward value^5,9^. Pupil response has been shown to increase following changes in reward contingencies and to reflect both error-related feedback and uncertainty^5,30^. Consistent with this, pupil dilation was found to vary with reinforcement history, being larger for highly reinforced cues early in learning and for partially reinforced cues at the end of the experiment, suggesting sensitivity to changing expectations over time^30^. Viewed together, these findings indicate that LC-NE activity reflected in pupil dilation is part of the organism’s response to aspects in the environment that drive feedback learning, particularly valence, and surprise. Although pupillometry has not yet been applied to study decision-making or feedback learning in individuals with DS, FXS, or WS, there is some evidence for altered pupil responses to emotionally valenced or social information in FXS and WS populations^22,31–33^ and during attention processing in DS^23^.

Where individuals fixate their gaze before making a choice can predict their decision-making^34–36^, which has been observed across development from early childhood^37^ to older adulthood^38^. Longer looking times toward an option generally increase the likelihood of selecting it, reflecting both preference and goal-directed attention^34,39^. While longer looking times increase the probability of choosing a specific option, the individual variability regarding this relationship is high^40^. Visual attention thus plays a key role in feedback-based decision-making, particularly in distinguishing between task-relevant and task-irrelevant stimuli^5^.

This makes it particularly pertinent to examine populations in which attention is known to be atypical, such as individuals with DS, FXS, and WS. Most existing work has focused on visual attention in social contexts such as social preference, theory of mind tasks, and face perception, or on basic attention paradigms. Across these studies, atypical visual attention has been identified in DS^15^, FXS^41^, and WS^42,43^. However, less is known about how gaze supports non-social decision-making, where attentional mechanisms may operate differently.

The current study aimed to examine behavioral performance and pupil dilation to feedback in individuals with ID with a known genetic etiology, through a reward contingency reversal task combined with eye tracking. Feedback was presented with varying feedback valence (win, interpreted as positively valenced, and loss, interpreted as negatively valenced), both visually and aurally. The reward followed a deterministic reward schedule. We specifically aimed to address the following research questions:

1. Do feedback learning behaviors (the probability of shifting action after negative feedback and repeating actions after positive feedback) differ between ID and TD, and between specific syndromes?
2. Does pupil dilation to feedback differ between ID and TD, and between specific syndromes?
3. Does gaze allocation to stimuli associated with rewarded as compared to non-rewarded actions differ between ID and TD, and between specific syndromes?
4. Are feedback learning behaviors predicted by pupil dilation and gaze allocation?

This study took a mainly exploratory approach due to the limited research on feedback processing in individuals with rare genetic syndromes associated with ID. Here, we primarily investigated differences in pupil dilation, gaze allocation, and decision-making relative to the patterns expected in TD individuals. For pupil dilation and its relation to feedback, we expected an increased pupil dilation following losses, reflecting heightened arousal to error-related feedback and surprise in TD individuals. We anticipated that individuals with FXS and WS would show an altered response pattern, consistent with prior findings of altered physiological reactivity to emotionally valenced information in FXS and WS.

## Results

### Descriptive data

Demographic data are shown in Table 1. Normality was assessed through visualization. A Bayesian linear regression was conducted to investigate differences in age and Global Adaptive Functioning (GAF) distributions. To examine differences in sex distribution across syndrome groups, a Bayesian logistic regression was used. All groups showed differences in age distribution, with the DS group having older participants than the other groups, and the FXS group generally having younger participants. The TD group had older participants than the WS group. The sex distribution among syndrome groups varied across the groups. As expected, a higher proportion of male participants was seen in the FXS group compared to all other groups. The DS group also had a greater proportion of male participants than the TD group, but did not differ from the WS group. Finally, the TD group had a lower proportion of male participants compared to the WS group. To address the uneven distributions of age and sex between groups, these variables were added as predictors to all models to investigate whether age or sex affected the results. For a visualization of the age distribution in the different groups, see Supplementary Fig. S4 in the Supplementary material. Model comparisons revealed that they did not have an effect on any of the outcome variables and were therefore not included as predictors in the final models. For the full result and model comparison, see Supplementary Tables S1–3 in the Supplementary material. FXS and DS groups did not differ in their estimated mean GAF scores, while the WS group showed a higher estimated mean GAF score (Table 1). Regarding differences in ID severity range distributions across the syndrome groups, Bayesian contingency analyses were conducted using the BayesFactor package in R^44^. Specifically, pairwise comparisons between the syndromes showed moderate to very strong evidence that the proportion of individuals within each severity category did not differ for DS and FXS (DS-FXS: BF01 = 3.23) and WS and FXS (FXS–WS: BF01 = 484.02), but the evidence was inconclusive for a similar distribution between WS and DS (DS–WS: BF01 = 2.13), meaning that their distributions of severity range might differ. In other words, the pattern of ID severity was mainly comparable across the syndrome groups. Group-level descriptive statistics for task performance measures are reported in Table 2.

**Table 1:** Sample characteristics are presented in mean values and standard deviations

	DS (*n* = 13)	FXS (*n* = 13)	WS (*n* = 27)	TD (*n* = 56)	Group differences
	**M (SD)**	**M (SD)**	**M (SD)**	**M (SD)**	
Age, years	34.98 (14.83)	21.46 (12.32)	22.68 (13.3)	24.99 (10.84)	DS > TD > WS > FXS
Age range, years	6–58	10–50	6–57	6–60	
Sex, f/m	4/10	1/12	11/17	33/24	TD > WS | DS > FXS
GAF score	58 (7)	57 (3)	70 (10)	–	WS > FXS | DS
Pupil baseline (mm)	2.74 (0.62)	3.10 (0.45)	3.15 (0.38)	2.83 (0.32)	

Demographic data for the sample. Standard deviation in parentheses.

The mean GAF scores are from eleven individuals with WS, nine individuals with DS, and eleven individuals with FXS.

*M* mean, *SD* Standard deviation, *DS* Down Syndrome, *FXS* Fragile X Syndrome, *TD* Typically Developed, *WS* Williams Syndrome, *GAF* Global Adaptive Functioning.

**Table 2:** Descriptive statistics for the final score and proportion of correct choices across groups

	DS	FXS	WS	TD
	**M (SD)**	**M (SD)**	**M (SD)**	**M (SD)**
Final score (points)	113 (4.05)	116 (1.82)	117 (1.19)	117 (1.27)
Proportion of switches	0.63 (0.48)	0.46 (0.50)	0.34 (0.47)	0.30 (0.46)
Proportion switches after a win	0.57 (0.00)	0.40 (0.00)	0.29 (0.00)	0.27 (0.00)
Proportion switches after a loss	0.83 (0.00)	0.85 (0.00)	0.91 (0.00)	0.80 (0.00)

The proportion of switches refers to the proportion of change in responses across trials. The final score refers to the mean final score on the last trial of the experiment. Proportion optimal choices refer to the proportion of trials where the participant made the optimal choice based on a previous trial where they received a point.

*M* mean, *SD* Standard deviation, *DS* Down Syndrome, *FXS* Fragile X Syndrome, *TD* Typically Developed, *WS* Williams Syndrome.

### Preliminary analysis

We fitted a model with stimulus type added as a predictor to investigate whether it affected the outcomes; it did not (see Supplementary Tables S10–S12 and Supplementary Fig. S5 in the Supplementary Material for full results).

### Does feedback valence influence the likelihood of changing responses across syndromes?

In the model, TD and loss feedback serve as reference categories, meaning that all group and feedback effects are interpreted relative to TD and loss feedback. As expected (Table 3), participants were less likely to change their responses following a win compared to a loss (*b* = −3.04, 95% CI = [−3.38 −2.71], BF > 150, *pd* = 1). As can be seen in Table 3, DS, FXS, and WS were more likely to change response after losses than TD, although robust evidence for a difference from TD was only found in the WS group. Both WS and DS differed from TD in their likelihood to change response *after wins*, but in opposite directions. DS were more likely to change response after wins (*b* = 1.47, 95% CI = [0.74, 2.18], BF > 150, *pd* = 1), whereas WS were less likely (*b* = -0.87, 95% CI = [−1.51, −0.23], BF = 12.79, *pd* = 1). For FXS, there was inconclusive evidence for an interaction effect between syndrome and previous feedback (*b* = 0.39, 95% CI [−0.33, 1.09], BF = 0.67, *pd* = 0.86).

**Table 3:** Fixed effects from the GLMM for the likelihood of changing response after feedback

Fixed effects	Estimate [95% CI]	SD	OR [95% CI]	BF10	BF01	pd	*p*-value
Intercept (TD + Loss)	**1.29 [1.04, 1.55]**	0.13	**3.64 [2.84, 4.70]**	**>999**	<0.00	1	<0.001
Prev. feedback (Win)	**−3.04 [−3.38, −2.71]**	0.17	**0.05 [0.03, 0.07]**	**>999**	<0.00	1	<0.001
DS	0.32 [−0.17, 0.81]	0.25	1.37 [0.84, 2.26]	1.09	0.92	0.90	0.435
FXS	0.37 [−0.13, 0.89]	0.26	1.44 [0.88, 2.44]	1.39	0.72	0.92	0.396
WS	**0.77 [0.28, 1.27]**	0.25	**2.15 [1.32, 3.57]**	**63.24**	0.02	1	0.011
Prev. feedback* DS	**1.47 [0.74, 2.18]**	0.37	**4.37 [2.11, 8.84]**	**689.15**	0.01	1	<0.001
Prev. feedback* FXS	0.39 [−0.33, 1.09]	0.36	1.47 [0.72, 2.98]	0.67	1.49	0.86	0.528
Prev. feedback* WS	**−0.87 [−1.51, −0.23]**	0.33	**0.42 [0.22, 0.79]**	**12.79**	0.08	1	0.03

The intercept represents TD at negative feedback on the previous trial.

Estimates are presented in logit values.

Bold indicates that 0 is not included in the 95% credible interval.

*TD* Typically Developed, *DS* Down Syndrome, *FXS* Fragile X Syndrome, *WS* Williams Syndrome, *BF* Bayes factor, *CI* Credible interval, *OR* Odds ratio, *pd* Probability of direction.

**Fig. 1:**
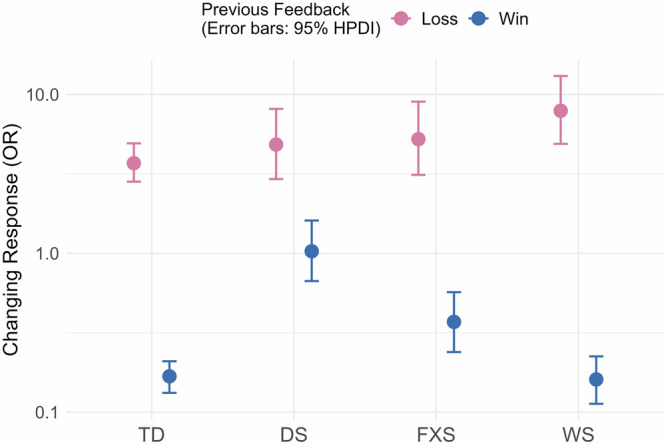
Likelihood of changing response after feedback on the previous trial across syndrome*.* Within-group contrasts presented in odds ratios for changing the response after feedback on the previous trial across the groups. Error bars represent the 95% highest probability density interval. TD Typically Developed, DS Down Syndrome, FXS Fragile X Syndrome, WS Williams Syndrome.

Follow-up pairwise comparisons were conducted (Fig. 1). Within-group comparisons showed that all groups were more likely to change their response after losses than wins (TD: OR = 20.97, 95% HPD [14.73, 28.92]; DS: OR = 4.78, 95% HPD [2.11, 8.71]; FXS: OR = 14.23, 95% HPD [6.55, 25.89]; WS: OR = 49.56, 95% HPD [25.61, 84.16]). To further explore differences between the syndromes, follow-up pairwise comparisons were conducted. The results showed that after positive feedback (win), DS individuals were more likely to change their response than both FXS individuals (OR: 2.8, 95% HPD [1.08, 5.41]) and WS individuals (OR: 6.59, 95% HPD [2.93, 11.96]), as indicated by the HPD interval not covering 1. FXS individuals were more likely to change response after wins than WS individuals (OR: 2.34, 95% HPD [1.03, 4.16]). Altogether, these results suggest that all groups were sensitive to feedback valence but that DS and FXS individuals showed a higher tendency to change responses after positive feedback, while WS individuals were less likely to change their responses, indicating more stable behavior after wins.

#### Do individuals with higher adaptive functioning adapt their behavior more effectively after feedback?

We examined whether an individual’s GAF score was related to sensitivity to feedback by using the random effects from the response change model within a Bayesian linear regression. The findings indicated no reliable link between sensitivity to feedback and GAF score (*b* = −0.00, 95% CI [−0.03, 0.02], BF = 0.01, *pd* = 0.57). These results should be interpreted with caution due to the limited sample size and low variability in GAF scores.

### Does pupil dilation to feedback valence differ between the groups?

**Fig. 2:**
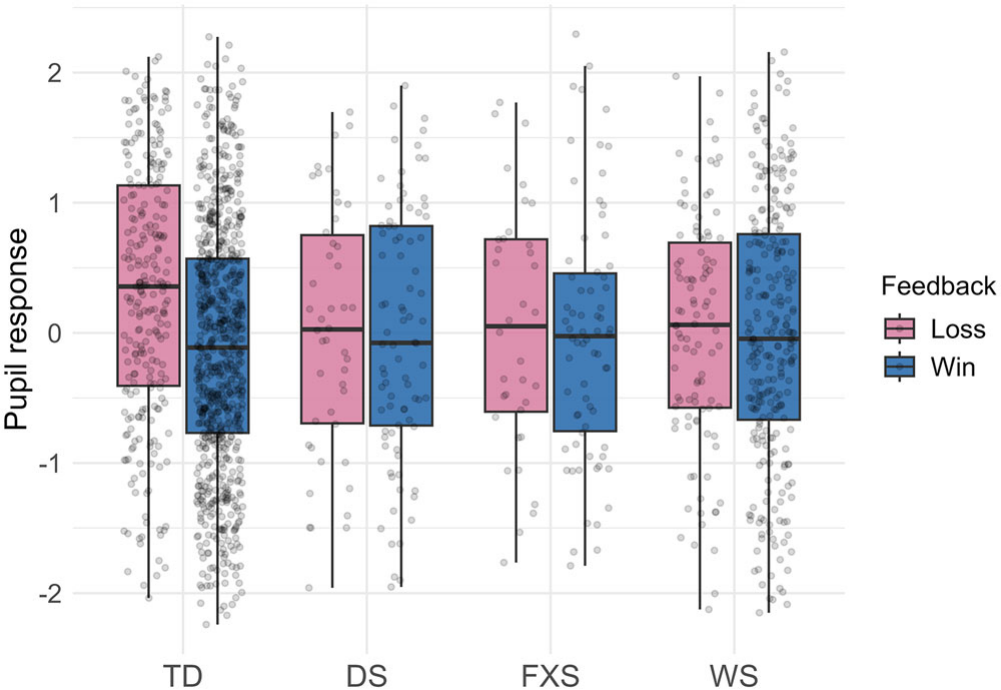
Pupil response in relation to feedback valence across the groups. The figure illustrates overall pupil response to feedback valence across the four groups. Pupil response refers to the change in mm (z-transformed) in relation to baseline. Individual datapoints represent individual trials. TD Typically Developed, DS Down Syndrome, FXS Fragile X Syndrome, WS Williams Syndrome.

In the model, TD and loss feedback serve as reference categories, meaning that all group and feedback effects are interpreted relative to TD and loss feedback. As can be seen (Table 4), pupil dilation were, overall, smaller following wins compared to losses (*b* = -0.37, 95% CI [−0.50, −0.23], BF > 150, *pd* = 1) and the pupil dilation decreased throughout the experiment (*b* = -0.01, 95% CI [−0.02, −0.01], BF < 0.01, *pd* = 1). DS, FXS, and WS had smaller pupil dilation to losses compared to TD, although robust evidence for a difference from TD was only found in the WS group (Table 4). For WS, there was moderate evidence for an interaction effect between syndrome and feedback (*b* = 0.34, 95% CI [0.09, 0.59], BF = 6.66, *pd* = 1), indicating that feedback valence influenced pupil responses differently in WS compared to TD. This effect was not evident for the FXS or DS groups. To further characterize this difference between WS and TD, follow-up pairwise comparisons were conducted (Fig. 2). For the TD group, pupil dilation were larger following negative feedback (losses) compared to positive feedback (wins) (*b* = 0.37, 95% HPD [0.23, 0.5]). In contrast, the WS group showed no reliable difference in pupil response between negative and positive feedback (*b* = 0.03, 95% HPD [−0.19, 0.24]). This suggests that for the WS group, feedback valence may have no reliable effect on pupil dilation. Overall, it suggests that individuals with WS did not show the expected loss-driven pupil dilation evident in TD individuals. For those with FXS and DS, the evidence for a different pupil response to feedback compared to TD or WS remains inconclusive.

**Table 4:** Results for pupil dilation in relation to feedback valence across the groups

Fixed effects	Estimate [95% CI]	SD	BF10	BF01	pd	*p*-value
Intercept (TD + Loss)	**0.45 [0.31, 0.59]**	0.07	**<0.00**	**>999**	1	<0.001
Feedback (Win)	**−0.37 [−0.50, −0.23]**	0.07	**>999**	**<0.00**	1	<0.001
DS	−0.23 [−0.51, 0.06]	0.15	0.68	1.47	0.93	0.314
FXS	−0.23 [−0.55, 0.08]	0.16	0.62	1.61	0.92	0.372
WS	**−0.25 [−0.47, −0.04]**	0.11	**2.58**	**0.39**	0.99	0.068
Trial	**−0.01 [−0.02, −0.01]**	0.00	**<0.00**	**>999**	1	<0.001
Feedback*DS	0.29 [−0.06, 0.65]	0.18	0.95	1.05	0.94	0.270
Feedback*FXS	0.28 [−0.10, 0.66]	0.19	0.79	1.27	0.92	0.386
Feedback*WS	**0.34 [0.09, 0.59]**	0.09	**6.66**	**0.15**	1	0.030
Sigma	0.96 [0.92, 0.99]	0.02				

Intercept refers to the TD group and negative feedback.

The estimates are presented as posterior means, along with 95% credible intervals, in z-values.

Bold indicates that 0 is not included in the 95% credible interval.

*DS* Down Syndrome, *FXS* Fragile X Syndrome, *TD* Typically Developed, *WS* Williams Syndrome, *BF* Bayes factor, *CI* Credible interval, *pd* Probability of direction.

### Does pupil dilation predict the likelihood of changing response?

To examine whether pupil response to feedback predicts the likelihood of a change in response across the groups, we fitted a GLMM for each syndrome using the loss-feedback trials only, with varying intercepts for participants. Pupil dilation affected response behavior in TD participants, suggesting that smaller pupil dilation was associated with a higher likelihood of response change (*b* = −0.51, 95% CI = [−0.85, −0.18], BF = 9.64, *pd* = 1). The opposite pattern was observed in FXS individuals, where a larger pupil dilation was associated with a higher likelihood of changing response on loss trials (*b* = 1.24, 95% CI = [0.02, 2.63], BF = 2.80, *pd* = 0.98). For WS and DS, there was no robust evidence for an effect regarding pupil dilation predicting response change on loss trials (DS: *b* = 0.43, 95% CI = [−0.52, 1.45], BF = 0.45, *pd* = 0.81; WS: *b* = −0.33, 95% CI = [−1.16, 0.46], BF = 0.36, *pd* = 0.79).

### Does feedback influence gaze allocation?

In the model, TD and loss feedback serve as reference categories, meaning that all group and feedback effects are interpreted relative to TD and loss feedback. As can be seen (Table 5), participants overall looked longer at the option that was associated with a win compared to a loss (*b* = 0.43, 95% CI [0.23, 0.63], BF > 150, *pd* = 1) and as the task progressed, participants looked less at the previously chosen option (*b* = −0.02, 95% CI [−0.02, −0.01], BF < 0.01, *pd* = 1). FXS and WS looked less at the previously chosen option compared to DS and TD, although no evidence for a difference from TD was found for any of the syndrome groups. In addition, there was no evidence for an interaction effect between the previously chosen option and gaze allocation for any of the syndrome groups. Together, these results suggest that all of the groups are more likely to look at the previously chosen option associated with a win compared to a loss.

**Table 5:** Results of the Bayesian GLMM on fixations based on previous feedback

Fixed effects	Estimate [95% CI]	SD	OR [95% CI]	BF10	BF01	pd	*p*-value
Intercept (TD + Loss)	**−1.12 [−1.31, −0.94]**	0.09	**0.32 [0.27, 0.39]**	**12.04**	**0.08**	1	<0.001
Prev. feedback (Win)	0.43 [0.23, 0.63]	0.10	**1.53 [1.25, 1.88]**	**332**	**<0.01**	1	<0.001
DS	0.01 [−0.29, 0.31]	0.16	1.01 [0.74, 1.37]	0.15	6.67	0.54	0.989
FXS	−0.24 [−0.63, 0.14]	0.20	0.78 [0.53, 1.15]	0.41	2.44	0.89	0.478
WS	−0.11 [−0.38, 0.15]	0.14	0.90 [0.68, 1.16]	0.19	5.26	0.79	0.728
Trial	**−0.02 [−0.02, −0.01]**	0.00	**0.99 [0.98, 0.99]**	**<0.01**	**>999**	1	0.001
Prev. feedback *DS	0.02 [−0.45, 0.48]	0.23	1.02 [0.64, 1.61]	0.23	4.37	0.53	0.997
Prev. feedback * FXS	0.04 [−0.49, 0.57]	0.27	1.04 [0.62, 1.78]	0.27	3.70	0.56	0.950
Prev. feedback * WS	0.04 [−0.32, 0.41]	0.19	1.04 [0.72, 1.50]	0.19	5.26	0.58	0.991
Phi	0.38 [0.35, 0.40]	0.01					

The intercept refers to the TD group and the negative previous feedback.

Bold indicates that 0 is not included in the 95% credible interval.

Estimates are presented in logit values and are posterior means.

*TD* Typically Developed, *DS* Down Syndrome, *FXS* Fragile X Syndrome, *WS* Williams Syndrome, *BF* Bayes factor, *CI* Credible interval, *OR* Odds Ratio, *pd* Probability of direction, *SD* Standard deviation.

To address the preregistered research question of whether gaze allocation predicts response change, we conducted the analysis despite the absence of an apparent group difference in gaze allocation. The results are presented in the Supplementary Material (Supplementary Table S13). An exploratory analysis of fixation on the center AOI revealed no evidence that WS or FXS participants differed from TD participants, while participants with DS spent less time fixating on the center AOI than TD participants (See Supplementary Material).

## Discussion

Individuals with ID, including those with DS, FXS, and WS, often show learning difficulties, but we know little about feedback processing in these conditions. While pupil dilation is well-established as an index of surprise and error feedback in typical development^9,29^, research on individuals with these syndromes has mainly focused on pupil response to social stimuli^20,22,33^. Our study extends this work by examining feedback processing in a non-social reinforcement learning task. Across all groups, behavioral results indicated that feedback learning took place to a high degree. Participants tended to stay with the positive feedback options and switch after receiving negative feedback. WS individuals exhibited a feedback learning pattern similar to TD individuals. Importantly, despite these feedback learning similarities, the physiological responses of WS individuals differed from those of TD individuals. Whereas TD individuals exhibited the expected larger pupil dilation to negative feedback compared to positive feedback^9,29^, WS individuals showed no difference, suggesting an atypical arousal response to feedback. Specifically, WS individuals had attenuated responses to losses, indicating hypoarousal to negative feedback. Individuals with DS and FXS exhibited greater variability in their feedback learning, being more likely to switch options after positive feedback, although these results should be interpreted with caution due to the smaller sample sizes. Gaze allocation supported the behavioral results, where all groups had a higher likelihood of looking at the previously rewarded option more than the loss option, indicating attention to relevant information. Taken together, these results reveal a dissociation in WS between intact behavioral feedback learning and altered physiological feedback processing, highlighting atypical arousal regulation to feedback.

In line with our hypothesis, the pupillary responses to feedback differed between TD and WS individuals, although their feedback learning responses during the task were comparable. Specifically, WS individuals did not demonstrate the expected loss-driven pupil dilation, suggesting that their feedback processing may rely on different underlying processes. Attenuated physiological responses, or *hypoarousal*, have been previously observed in WS, particularly in response to social information^20,33,45^, along with a lack of arousal synchronization (i.e., absent pupil contagion)^32^. Hypoarousal has been proposed as one explanation for the various social interaction difficulties often observed in WS, such as prolonged attention to faces, difficulties modulating attention, and the resulting heightened interest in social interactions^43^. Consistent with a broader hypoarousal profile, reduced habituation to both social and non-social visual stimuli has been reported in individuals with WS^46^. Combined with previous research regarding reduced amygdala activation to fearful faces^47^, this pattern was interpreted as reflecting reduced arousal to in WS. Interestingly, this pattern was not observed with non-social images^33^. The current findings extend this pattern to a non-social learning context. Furthermore, individuals with WS have been shown to exhibit higher amygdala activation for positive facial expressions, such as happy faces^47^, and they tend to learn more from social feedback compared to non-social (e.g., images of monetary rewards)^25^. These findings, combined with our findings in this study, emphasize the importance of considering both social and non-social contexts when studying feedback learning and physiological responses in individuals with WS. In the current study, we only used non-social feedback. Future research should directly compare social and non-social feedback types to determine whether arousal in a learning context in WS is domain-specific or reflects a broader alteration in feedback processing.

Larger pupil contraction has been observed in adults with autism in response to receiving rewards. Nevertheless, there was no difference in pupil responsiveness between the two different feedback anticipation phases^7^. The authors also reported a difference in pupil responses to various types of feedback, with social feedback producing enhanced responses compared to monetary and neutral feedback across all participants. For future research, it would be interesting to investigate the entire feedback process (anticipating reward and receiving feedback) in individuals with WS using various types of feedback to see if a similar pattern can be observed. For the DS and FXS groups, however, the evidence was inconclusive. Although both groups showed smaller pupil dilation to losses than TD individuals, the uncertainty in the estimates was large, and neither group demonstrated a reliable interaction between feedback valence and pupil dilation. Thus, unlike WS, we cannot draw firm conclusions about altered feedback processing in DS or FXS.

All groups successfully adapted their choices based on feedback, indicating that they understood the task and could apply a win-stay/lose-shift principle. This confirms that the simplified reward contingency reversal task was feasible for individuals with ID in the mild to moderate severity range and that performance was not limited by comprehension or task demands. The lack of major group differences in feedback learning is encouraging, as it suggests that observed differences in physiological responses, like pupil dilation, reflect a genuine variation in feedback processing rather than task difficulty. In real-world contexts, individuals with DS, FXS, and WS often experience learning difficulties, yet the current findings show that when task demands are clearly structured and feedback is consistent, on average, they can adapt effectively. Although all groups showed that they could adapt after feedback, individuals with DS and FXS were more variable in their learning strategies. That is, they were more likely to switch responses after feedback. While the group sizes for DS and FXS were small, these results align with previous research showing that individuals with DS experience more difficulties with executive functions such as working memory, shifting, task monitoring, and planning^48,49^, while individuals with FXS show challenges with inhibitory control^50–52^. Such difficulties are not unique to these syndrome groups, as challenges in executive functioning have also been reported in WS^53,54^. Overall, these results support the use of this paradigm in ID populations and provide a robust foundation for examining syndrome-specific differences in physiological feedback processing.

These behavioral results indicate that differences in physiological responses cannot be attributed to task misunderstanding or atypical attention allocation. Consistent with this, gaze allocation did not differ between the groups, in that all groups allocated their attention toward the option that had previously been rewarded. Generally, longer looking times on a specific option increase the likelihood of choosing that option^40,55^, and attention tends to be biased toward more valuable options^35^. However, the relationship between gaze and choice is weaker in a two-alternative task compared to a three-alternative task^40^. In sum, even though WS individuals attended to the rewarding option and adapted their behavior similarly to TD individuals, they showed no difference in pupil dilation to negative compared to positive feedback. These findings strengthen the interpretation that the group differences in pupil dilation, particularly for WS, reflect distinct underlying feedback processing mechanisms, rather than general performance or attention-related factors. Future research could build on this by employing more complex experimental paradigms to further understand the mechanisms driving these physiological differences.

This study has limitations that should be acknowledged. First, the data were collected using two different eye trackers with different sampling rates. Although calibration and data processing procedures were consistent across systems, hardware differences may have introduced some measurement variability. However, this potential variability is unlikely to account for the observed group differences, as the two systems were used across all groups rather than being linked to any specific group. Moreover, sample sizes were uneven across groups and rather small in both the DS and FXS groups (*n* = 13). Combined with the heterogeneity of these groups, including variation in age and co-occurring conditions, this may have reduced statistical power for some comparisons. In particular, differences in cognitive and behavioral characteristics among participants may have contributed to the observed variability in feedback learning. However, such heterogeneity is common in genetic syndromes associated with ID and represents an important aspect to consider for these populations. Future studies with larger sample sizes are needed to verify these findings and determine their generalizability. Additionally, we had limited measures of different cognitive abilities, such as general intelligence or reasoning (IQ), which restricted the ability to link feedback learning to specific cognitive profiles. Without IQ measures, we cannot rule out that the presence of ID itself, rather than syndrome-specific mechanisms, contributes to the observed differences between TD and WS groups in pupil response to feedback valence. Conclusions about syndrome-specific profiles may therefore be premature, as we cannot disentangle ID-related effects from WS-distinct patterns. We note that adding IQ as a covariate is debated in neurodevelopmental conditions research, as it may remove meaningful variance related to the disorder itself^56^. Future research with a more comprehensive cognitive assessment will be needed to better disentangle these effects. The current task was intentionally simplified to ensure feasibility for individuals with ID in the mild to moderate range, and most participants demonstrated a clear understanding of the principles for succeeding (lose-shift/win-stay strategy). The lack of observable difficulties in adapting to changing reward contingencies likely reflects this simplicity of the task rather than the absence of cognitive flexibility difficulties. Future research could therefore use more complex learning paradigms to better capture differences in flexibility and adaptability after feedback within genetic ID populations. Extending this research by examining how various types of feedback and rewards influence learning across genetic syndrome populations may provide deeper insight into the underlying arousal regulation and cognitive mechanisms underlying feedback learning. Given the relatively small and heterogeneous syndrome groups, the findings should be considered preliminary and interpreted with appropriate caution. Nevertheless, they provide an initial characterization of feedback learning across the syndrome groups and may help inform future research in larger samples.

In sum, this study reveals a distinct feedback-processing profile in WS and suggests that similar patterns may also be present in DS and FXS. All groups successfully adapted their behavior, demonstrating that the task effectively captured feedback learning in individuals with a genetic syndrome associated with ID. Crucially, WS individuals showed atypical pupil dilation to feedback despite performing at the same behavioral level and allocating gaze similarly to TD individuals. This dissociation indicates an altered physiological response to feedback that cannot be explained by differences in learning success or attentional focus and is consistent with a hypoarousal profile previously reported in WS. In contrast, DS and FXS participants showed more variable learning strategies, although these findings should be interpreted cautiously, given the heterogeneity of the groups. Furthermore, we cannot draw firm conclusions about altered pupil reactivity to feedback in DS or FXS. Together, these findings suggest that pupillometry can be useful for identifying differences in physiological responses to feedback that are not apparent from behavioral performance alone.

## Methods

### Participants

The final sample consisted of 109 individuals (TD *n* = 56; DS *n* = 13; FXS *n* = 13; WS = 27), but differed slightly across the different analyses. Participants were assessed between 2022 and 2025. Data was collected at the laboratory, at facilities near the participants’ assisted living accommodations, and at family gatherings organized by patient organizations. After the session, participants received a voucher for a cinema ticket. Participants’ task understanding was assessed using the proportion of trials in which they made the optimal choice, and those with a proportion of correct decisions below chance level (i.e., <0.6) were excluded from all analyses (DS: *n* = 2, FXS: *n* = 0, TD: *n* = 1, WS: *n* = 1). Genetic diagnosis was confirmed through medical records. See Table 1 for demographic data.

#### TD children and adults

Initially, 58 individuals were recruited via an advertisement on social media and a database for people who had expressed interest in participating in research. Participants older than 60 years of age (*n* = 1) were excluded from the analysis to match the age range of the participants in the syndrome groups. One individual was excluded from the analyses because their mean proportion of optimal choices was below 0.6. Two individuals did not provide valid gaze or pupil data and were therefore excluded from those analyses. Information regarding medical and psychiatric conditions was acquired through parental reports or self-reports. The final sample consisted of 56 participants aged 6–60 years for all the analyses.

#### DS

Fifteen individuals with DS aged 6–58 years participated in the study. Data regarding co-occurring conditions were obtained from 13 individuals through medical records. Co-occurring psychiatric conditions were autism (*n* = 2), attention-deficit/hyperactivity disorder (ADHD) (*n* = 2), language disorder (*n* = 2), depression (*n* = 2), sleep disorder (*n* = 1), and anxiety (*n* = 1). Co-occurring medical conditions were hypothyroidism (*n* = 6), heart condition (*n* = 1), and hearing impairment (corrected with hearing aids) (*n* = 8). Information regarding ID severity was available for ten individuals (mild, *n* = 1; moderate, *n* = 7; severe, *n* = 1; unspecified, *n* = 1); for the other three individuals, severity was not registered. Two individuals were excluded from all analyses because their mean proportion of optimal choices was below 0.6, indicating insufficient understanding of the task. The final sample for the behavioral data consisted of 13 participants. Two did not provide enough valid pupil data and were therefore excluded from those analyses, resulting in a final sample size of *n* = 11 in the pupil dilation analyses.

#### FXS

Fourteen individuals with FXS aged 10–50 years were recruited. One wanted to cancel participation due to difficulties with the task. This left a final sample size of 13 individuals in the analysis of behavioral data. Data regarding co-occurring conditions were obtained from twelve individuals through medical records. Co-occurring psychiatric conditions were autism (*n* = 10), ADHD (*n* = 5), and language disorder (*n* = 3). Co-occurring medical conditions were epilepsy (*n* = 3). Information regarding ID severity was available for ten individuals (mild *n* = 6, moderate *n* = 3, unspecified *n* = 1). For the other two individuals, severity was not registered. Two individuals did not provide enough valid pupil data and were therefore excluded from those analyses, resulting in a final sample of *n* = 11 in the analyses of pupil dilation.

#### WS

Twenty-eight individuals with WS aged 6–57 years old participated. Data regarding co-occurring conditions were obtained from 16 individuals through medical records. Co-occurring psychiatric conditions were autism (*n* = 2), ADHD (*n* = 2), tics disorder (*n* = 1), language disorder (*n* = 3), dyslexia (*n* = 1), and specific phobia (*n* = 2). Co-occurring medical conditions were hypothyroidism (*n* = 1), type 2 diabetes (*n* = 1), kidney condition (*n* = 2), and heart condition (*n* = 9). Information regarding ID severity was available in the medical records from 14 participants (mild *n* = 9, moderate *n* = 2, unspecified *n* = 1, no ID = 2). Five individuals had a research diagnosis from participating in a larger assessment, which included a diagnostic interview regarding psychiatric conditions and neuropsychological testing conducted by a clinical psychologist or a child and adolescent psychiatrist^57^. Co-occurring research diagnoses were autism (*n* = 1), ADHD (*n* = 1), depression (*n* = 1), panic disorder (*n* = 2), obsessive-compulsive disorder (OCD) (*n* = 1), generalized anxiety disorder (GAD) (*n* = 2), and specific phobia (*n* = 2) with three having a mild ID and two a moderate ID. One individual was excluded from the feedback analysis because their mean proportion of optimal choices was below 0.6, indicating insufficient understanding of the task. The final sample for the behavioral data consisted of 27 participants. Two individuals did not provide valid pupil data and were therefore excluded from those analyses, resulting in a final sample of *n* = 26 in the analyses of pupil dilation.

The DS, FXS, and WS participants were recruited through patient organizations and Swedish healthcare services.

### Experimental procedure

For identifying adaptive functioning in everyday life in the syndrome groups, the standard score of Global Adaptive Functioning (GAF) from the Swedish version of the Adaptive Behavior Assessment Scales (ABAS-3, third edition) (Harrison & Oakland, 2020) was used.

**Fig. 3:**
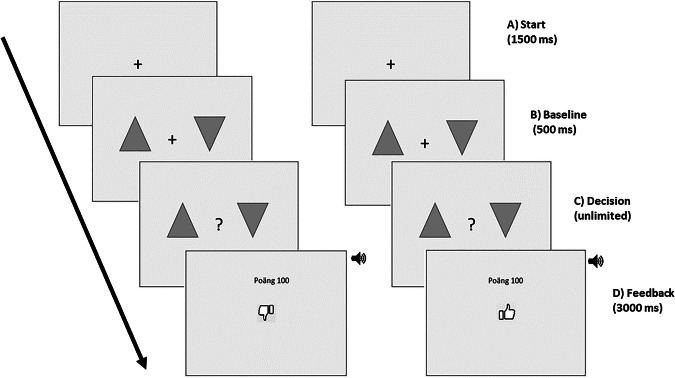
Illustration of a trial*.* Each trial began with a fixation cross displayed on the screen for 1500 ms **A**. A stimulus was then presented with a fixation cross at the center of the screen for 500 ms **B**. When “?” appeared in the middle of the screen, the participant could make a decision by pressing the corresponding key, with no time limit (**C**). After responding, participants received feedback on the screen for 3000 ms, accompanied by a sound indicating whether their response was correct or not **D**.

The reward contingency reversal task consisted of a maximum of 25 trials. See Fig. 3 for an example of a trial. Before the task, participants received instructions, both in written and oral form. Instructions were adjusted based on the participant’s receptive language skills. Following the instructions, two practice trials were conducted, one trial illustrated a win and the other illustrated a loss. The first trial was initiated either by the participant or the test leader by pressing a key on the keyboard. Each trial presented a pair of pictures, and participants were required to choose one of the options. The stimulus pairs included either two geometric figures with different orientations or two different faces presented in two randomized blocks. One option in each pair resulted in a win (1 point) and was indicated with a thumbs up and a specific sound, while the other option resulted in a loss (0 points) and was indicated with a thumbs down and a specific sound. The two sounds were matched for duration and volume. The stimulus pair was presented for 500 milliseconds (ms) before the participant could make a decision (by pressing a corresponding key on the keyboard). Feedback was presented for three seconds after the decision was made (henceforth *the feedback period*). After four consecutive correct responses (wins), the reward contingency reversed without warning, resulting in a loss. Each block consisted of a fixed number of trials in which the same correct response was repeated until the reward contingency reversed again. In total, the task included a total of five achievable blocks

### Eye-tracking apparatus and eye-tracking procedure

Before the start of the experiment, the calibration procedure was performed. Five calibration points were used, but for participants needing more assistance with instructions and attention, two calibration points were used (*n* = 12). Information regarding how many calibration points were used was missing for eleven individuals. Pupil size was recorded using two different corneal reflection eye-trackers: A Tobii Spectrum with a sampling rate of 1200 HZ (*n* = 79) and a Tobii X-120 with a sampling rate of 120 HZ for gaze position and 40 HZ for pupil data (*n* = 28) (Tobii Inc., Danderyd, Sweden). No chin-rest was used. In cases where the eye-tracker could not detect both eyes or when the calibration process was challenging for the participant, one eye was selected.

The preprocessing of the raw pupil data followed the guidelines recommended by^58,59^. Gaps in the data shorter than 250 ms were linearly interpolated. Pupil size changes ±3*median absolute distance (MAD) were removed, as were samples preceding and succeeding the sample with the unlikely change. The data was filtered using a moving average filter with a 200-ms window, which has also been used in infant research^60^. Pupil data recorded at 1200 HZ were downsampled to 40 HZ. For baseline correction, the subtractive baseline correction method was used, and the baseline was identified as 500 ms before the choice interval of each trial (Phase B in Fig. 3). Trials with fewer than 30% valid samples in baseline (DS: 45.6% of trials; FXS: 57.6%; WS: 28.4%; TD: 7.3%) and feedback period were excluded (DS: 33.8% of trials; FXS: 65.4%; WS: 21.9%; TD: 3%). Baselines and pupil dilations exceeding ±3*MAD for each individual were identified as outliers and removed from the analyses, which accounted for 6.5% of the data for baselines and 5.6% for the pupil responses.

Fixations were identified using the kollaR package in R^61^. Gaps shorter than 100 ms were interpolated and smoothed using a 15-ms moving average window. Saccades were identified using an I-VT filter algorithm with a velocity threshold of 30°/second. Fixations were defined as the intervals that occurred between saccades. The minimum fixation duration was set to 60 ms, and the minimum threshold for valid samples was set at 30%. Eye movement data were analyzed at the original sampling rate. Individuals with fewer than three trials without eye-tracking data were removed from the analyses (TD, *n* = 2; DS, *n* = 2; FXS, *n* = 2; WS, *n* = 1). Decisions regarding the processing of data were made before any statistical analyses.

### Pre-registered analysis plan

An analysis plan with the initial hypotheses, experimental variables, experimental paradigm, and planned statistical analysis was preregistered at Open Science Framework (OSF, https://osf.io/vpnhr/overview?view_only=b50f1f5ca1f34530b218a9a01f709347) during data collection and before looking at the data. Additional data from individuals with Smith-Magenis syndrome were originally planned to be included in the cross-syndrome comparison, but were removed from the analysis due to the limited number of participants we were able to recruit (*n* = 8). The pre-registered research question regarding reactions to surprise was not addressed, as the relatively small number of trials following shifts in reward contingency made it difficult to disentangle responses to surprise from loss-feedback effects.

### Statistical analysis

*Likelihood of changing response* was defined as whether the participants changed their response on the subsequent trial based on the outcome of the previous trial, coded as true/false. *Pupil dilation* refers to the change in pupil diameter (mm) from baseline (B in Fig. 3), during the feedback period (D in Fig. 3). *Gaze allocation* refers to the proportion of looking time on the different options (the two options and the center fixation) during the baseline and decision period (B and C in Fig. 3).

For the main analyses, Bayesian generalized linear mixed-effects models (GLMMs) or linear mixed-effects models (LMMs) were conducted. In contrast to traditional null hypothesis significance testing (NHST), Bayesian models combine prior beliefs (called *priors*) with the observed data to estimate updated beliefs (called *posteriors*) and the uncertainty around them, typically expressed as *credible intervals* (CI)^62^. The *Bayes factor* (BF) quantifies how much the data shifts our belief in favor of one hypothesis over another, and can provide evidence for, or against, the null hypothesis^63^. According to commonly used guidelines, a BF between 1 and 3 indicates inconclusive evidence, 3–10 indicates moderate evidence, and values above 10 indicate strong evidence for an effect. BF 0.33–0.1 indicated moderate evidence for the null hypothesis and BF < 0.1 indicates strong evidence for the null^64^. *Probability of direction* (pd) refers to the probability that an effect is consistently positive or negative, expressed as the proportion of the posterior distribution that has the same sign as the median^65^. *Highest posterior density interval* (HPDI) refers to the narrowest, or densest, interval containing the probability mass of the posterior distribution. For each effect, the beta estimate (posterior mean), 95% CI, BF, *pd*, and frequentist probability value (*p*-value) are reported. Evidence for effects was evaluated using BF, *pd*, and 95% CI. We interpreted BF > 3 or <0.33 as evidence for an effect, or for the null, and values in between as inconclusive. A *pd* ≥0.95 and a 95% CI that did not include zero were also taken as indicators of an effect.

All mixed-effects models were fitted at the trial level with random intercepts for participants to account for repeated measures and varying slopes for feedback or previous feedback. To address our research questions, the TD group was set as the reference group in all group comparison models, to allow comparisons relative to typical development. We used Markov Chain Monte Carlo (MCMC) sampling to estimate the posterior distributions of the parameters in the LMMs and GLMMs. The MCMC sampling was conducted using the brms package^66^ in R Studio^67^ with 10,000 iterations (half of them warm-up) over four chains and cores. The loo package^68^ was used for calculating the leave-one-out (LOO) cross-validation log score and for model comparison. For the follow-up pairwise comparisons, the emmeans package was used^69^. Visualizations were prepared using ggplot2^70^. To extract BF, *pd*, and *p*-value for the effects, the bayestestR package was used^65^. Since the current literature provides limited findings across genetic syndromes and feedback processing, we chose to apply weakly informative priors. Exponential priors were used for variance parameters and LKJ priors for correlation matrices. Prior predictive checks were performed for all models before fitting the observed data.

For the likelihood of changing response, a GLMM with a logit link function was fitted. The model examined whether the likelihood of changing response was influenced by feedback received on the immediately preceding trial (win vs. loss; henceforth referred to as *previous feedback*). Previous feedback (win vs. loss) and syndrome (TD, DS, FXS, WS) were included as predictors, along with their interaction, to test whether the effect of previous feedback on response change differed between groups. (The fitted model: Changed response (yes/no) ~ Previous feedback (loss/win) + syndrome (TD/DS/FXS/WS) + previous feedback (loss/win) * syndrome (TD/DS/FXS/WS) + (previous feedback| participant)).

For pupil dilation, a LMM was conducted to analyze whether the pupil dilation to feedback valence differs between the groups. Feedback (win vs. loss) and syndrome were used as predictors. Trial was added as a predictor to examine whether the pupil dilation would differ between the beginning and the end of the experiment. (The fitted model: Pupil dilation (mm) ~ feedback (loss/win) * syndrome + trial + (feedback|participant)).

To analyze whether pupil dilation to feedback predicted the likelihood of changing response, a GLMM with a logit link function was fitted, using only loss-outcome trials, including a random intercept for participant to account for repeated measures. The model was fitted separately for each syndrome group. (Fitted model: Changed response ~ pupil dilation + (1|id)).

To analyze whether feedback on the previous trial predicted the proportion of fixation time on the previously chosen option in the subsequent trial, and whether this relationship differed between groups, a GLMM with a Beta distribution and a logit link was fitted. Previous feedback and syndrome were included as predictors, along with their interaction. Trial was also added as a predictor. (Fitted model: proportion of fixation time on previously chosen option ∼ previous feedback + syndrome + previous feedback * syndrome + trial + (previous feedback|id)).

To analyze whether gaze allocation predicted the likelihood of changing response, a GLMM with a logit link function was fitted. The model tested whether this relationship varied by previous feedback, and was fitted separately for each syndrome group. (Fitted model: Changed response ~ previous feedback * proportion of fixation time on the previous choice + (1|id)).

For model comparisons and prior predictive checks on all outcome measures, see Supplementary Figs. S1–3 and Supplementary Tables S4, S6, S8 in the Supplementary material. For sensitivity checks, see Supplementary Tables S5, S7, S9 in the Supplementary material.

### Ethical approval and consent to participate

The study was conducted following the declaration of Helsinki and approved by the Regional Ethics Committee of Stockholm, Sweden (dnr 2018/1218-31 with subsequent amendments). Participants aged 15 years or above gave verbal and written consent to participate. Legal guardians of participating children gave written consent. For participants with an ID, a representative (relative or a legal representative) gave written and verbal consent. Informed assent was obtained from all participants who were able to assent.

## Supplementary information

Supplementary Information

## Data Availability

The data that support the findings of this study are available from the corresponding author (AH) upon reasonable request, following the ethics approval.

## Code availability

The underlying code for this study is available from the corresponding author (AH) upon reasonable request, following the ethics approval.
