# Supplementary material for: Comparing feedback learning and arousal responses in Down, Fragile X, and Williams syndromes
Source: NPJ Sci Learn. 2026 Jul 16;11:46. doi: 10.1038/s41539-026-00438-2 (PMC13377110; doi:10.1038/s41539-026-00438-2)
Supplement: Supplementary file 1 — Supplementary Information [file 41539_2026_438_MOESM1_ESM.pdf]

Supplementary Material

Prior Predictive Checks

For the likelihood to change response model, previous results on how cues impact learning ability in WS and DS found varying effects depending on mental age[1], and priors were weakly informed by those results. See Figure S1 for prior predictive distributions. For the pupil response model, where some previous evidence exists on TD individuals [2], the priors were based on these results. See Figure S2 for prior predictive distributions. For the fixation duration model, we based the priors on results from previous research regarding how feedback influences gaze behavior [3], where a moderate effect size was found for higher dwell time on correct options. See Figure S3 for prior predictive distributions.

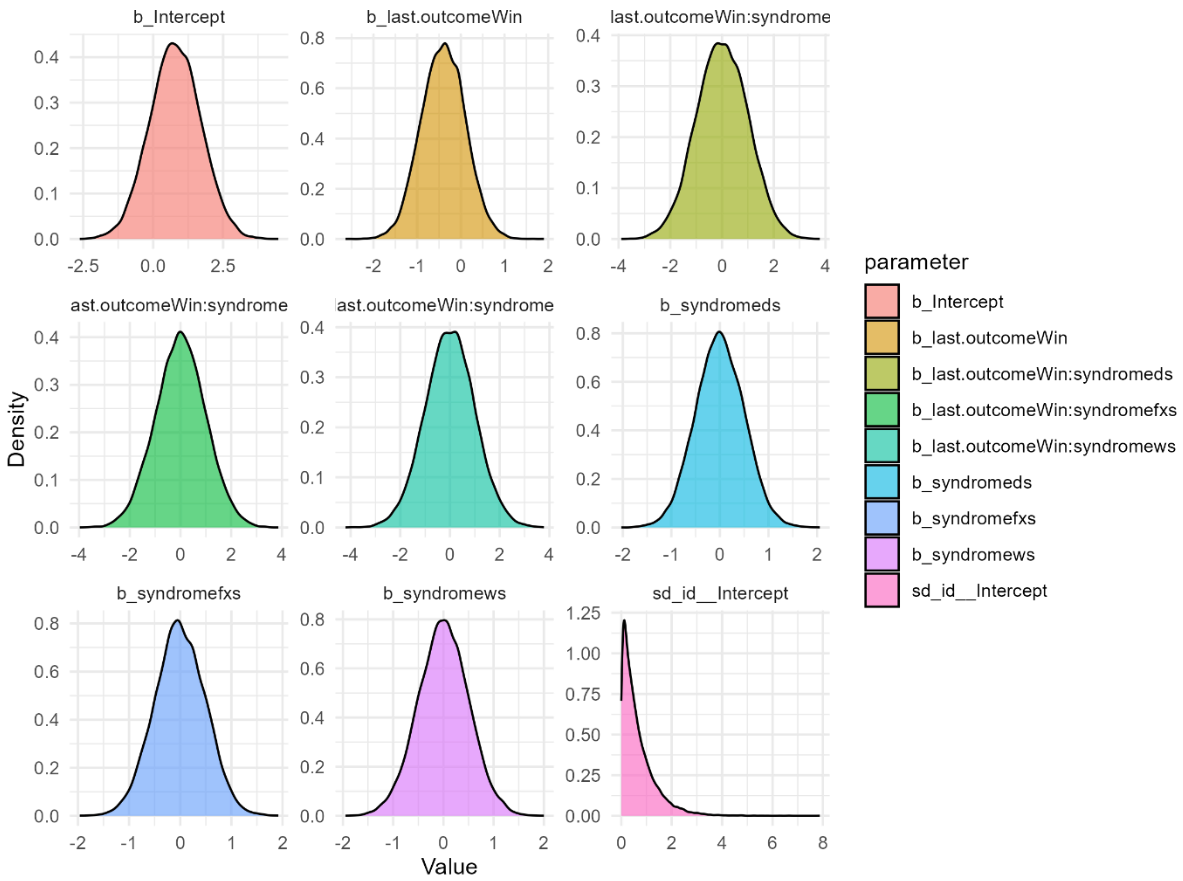

Figure S1. Prior predictive distributions for how feedback affects choice behavior on the subsequent trial. Prior predictive distributions for each parameter. SD_id_Intercept refers to the random effect. Fixed effects were assigned Normal priors (Intercept: Normal(0.5, 0.8); Previous feedback: Normal(-0.4, 0.5); Syndrome: Normal(0, 0.5); Previous feedback ×

Syndrome: Normal(0, 1)). Variance parameters were assigned Exponential(1.5) priors and the correlation matrix an LKJ(2) prior.

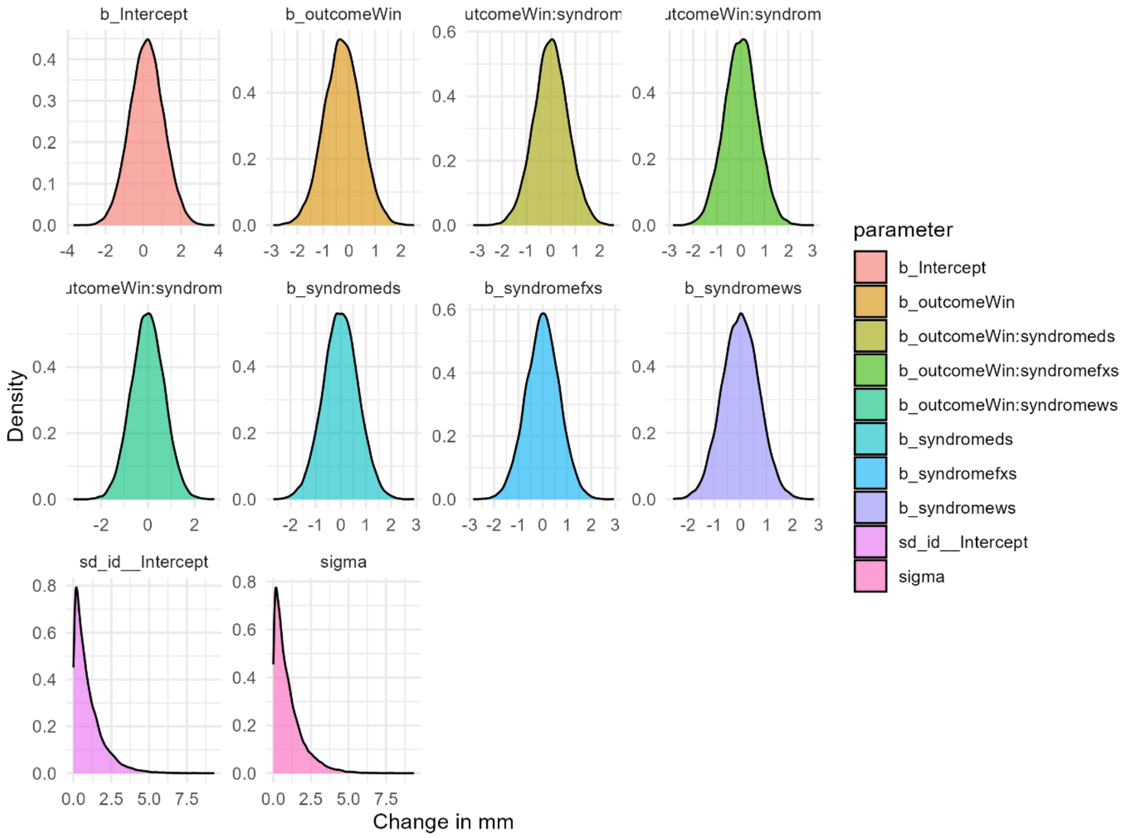

Figure S2. Prior predictive distributions for how previous feedback affects pupil response. Prior predictive distributions for each parameter. SD_id_Intercept refers to the random effect. Fixed effects were assigned Normal priors (Intercept: Normal(0, 0.7); Feedback: Normal(-0.25, 0.7); Syndrome: Normal(0, 0.7); Feedback × Syndrome: Normal(0, 0.7)). Variance parameters were assigned Exponential(1) priors and the correlation matrix an LKJ(2) prior.

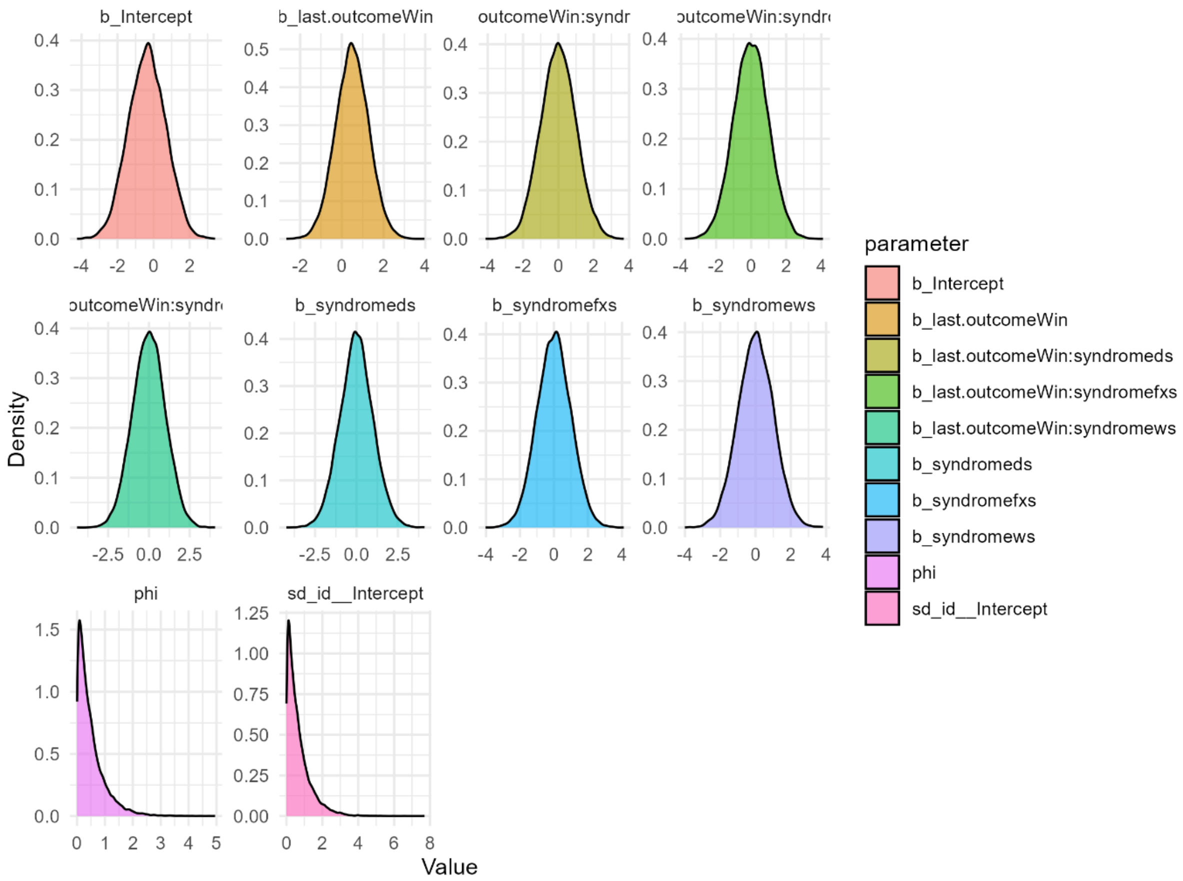

Figure S3. Prior predictive distributions for how feedback affects gaze behavior. Prior predictive distributions for each parameter. SD_id_Intercept refers to the random effect. Fixed effects were assigned Normal priors (Intercept: Normal(0, 0.8); Previous feedback: Normal(0.5, 0.8); Syndrome: Normal(0, 1); Previous feedback × Syndrome: Normal(0, 1)). Variance parameters were assigned Exponential(1.5) priors, phi exponential(2) and the correlation matrix an LKJ(2) prior.

Results

The age distributions of the different groups are shown in Figure S4.

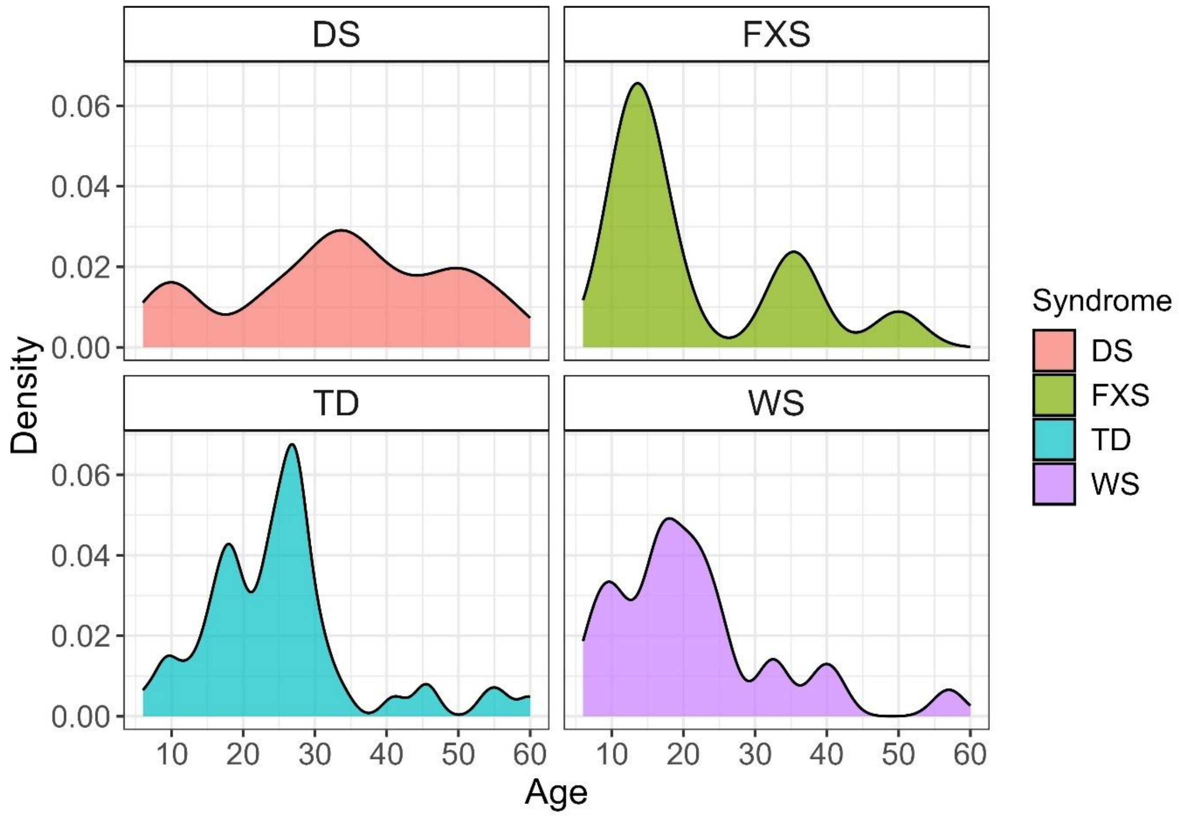

Figure S4. Density curves of the age distribution within the different groups. DS = Down Syndrome, FXS = Fragile X Syndrome, TD = Typically Developed, WS = Williams Syndrome.

The impact of age and sex on the outcome variables

Feedback valence and likelihood of changing response

Separate Bayesian GLMMs with age and sex as predictors on the likelihood of changing response (True/False) based on previously received feedback (loss/win) were conducted. A Bernoulli distribution with a logit link was used. All $\hat{R}$ were close to 1.00, which indicated that the chains had converged and were exploring the parameter space effectively. Three separate models were fitted. One null model (with no predictor), one with age as a predictor, and one with sex as a predictor. Model comparisons were then carried out using Leave-one-out cross-validation (LOO) in accordance with recommendations when dealing with weak priors [4]. All Pareto k estimates were deemed good ($k < 0.7$). As can be seen (Table S1), the similarities between the models were high, and neither age nor sex enhanced the model fit. Age and sex were therefore not included as predictors in the final model.

Table S1

Model comparison values for the likelihood of changing response based on previous feedback models

Model	Elpd LOO (SE)	P LOO (SE)	LOO IC (SE)	Elpd diff.	SE diff.
Null model	-1117.7 (29.6)	69.1 (2.3)	2235.3 (59.1)	0.0	0.0
Predictor Age	-1118.1 (29.6)	69.9 (2.4)	2236.2 (59.3)	-0.4	0.3
Predictor Sex	-1118.0 (29.6)	69.8 (2.4)	2236.1 (59.2)	-0.4	0.3

Note. Elpd = Expected log predictive density, SE = Standard error, P LOO = estimated effective number of parameters, LOO IC = Leave-one-out information criteria.

Feedback valence and pupil dilation

Separate Bayesian LMMs were fitted to analyze pupil response to feedback outcome (loss/win). All $\hat{R}$ were close to 1.00, which indicated that the chains had converged and were exploring the parameter space effectively.

For the model comparison, the same execution was used as for the feedback valence and choice behavior model. All Pareto k estimates were deemed good ($k < 0.7$). As can be seen in Table S2, differences in expected log predictive density (epld) were small and the standard error (SE) large, indicating sex and age did not improve model fit. The predictors are therefore not included in the final models.

Table S2

Model comparison values for feedback valence on pupil response models

Model	Elpd LOO (SE)	P LOO (SE)	LOO IC (SE)	Elpd diff.	SE diff.
Null model	-2252.3 (23.2)	3.7 (0.1)	4504.7 (46.4)	0.0	0.0
Predictor Age	-2253.4 (23.2)	4.7 (0.2)	4506.8 (46.4)	-0.2	-1.1
Predictor Sex	-2253.3 (23.2)	4.6 (0.1)	4506.8 (46.4)	-0.2	-0.9

Note. Elpd = Expected log predictive density, SE = Standard error, P LOO = estimated effective number of parameters, LOO IC = Leave-one-out information criteria.

Gaze allocation and previous feedback

Separate Bayesian GLMMs were fitted to analyze how gaze allocation (proportion of fixation time on the previously chosen option) was influenced by the feedback outcome (loss/win) from the previously conducted trial. A Beta distribution with a logit link was used. All $\hat{R}$ were close to 1.00, indicating that the chains have converged and were effectively exploring the parameter space.

For the model comparison, the same execution was used as for the feedback valence and the likelihood of changing response model. All Pareto *k* estimates were considered good ($k < 0.7$). As can be seen in Table S3, differences in epld were small and the SE large, indicating that sex and age did not improve model fit. The predictors were therefore not included in the final model.

Table S3

Model comparison values for feedback outcome on the previous trial and gaze allocation

Model	Elpd LOO (SE)	P LOO (SE)	LOO IC (SE)	Elpd diff.	SE diff.
Null model	10472.8 (333.3)	85.5 (3.2)	-20945.7 (666.7)	0.8	-0.1
Predictor Age	10472.8 (333.3)	85.6 (3.2)	-20945.5 (666.7)	0.0	0.0
Predictor Sex	10472.5 (333.3)	86.1 (3.3)	-20944.9 (666.7)	0.9	-0.4

Note. Elpd = Expected log predictive density, SE = Standard error, P LOO = estimated effective number of parameters, LOO IC = Leave-one-out information criteria.

Model Comparisons

Feedback valence and likelihood of changing response

Five separate models were fitted. One null model (with no predictor), one with only previous feedback as a predictor, one additive model (previous feedback and syndrome), one with an interaction term (previous feedback * syndrome), and one with varying slopes for previous feedback. Model comparisons were then carried out using the loo-package in R [4]. All Pareto *k* estimates were deemed good ($k < 0.7$). As can be seen (Table S4), the similarities between the models were high, but the model with the varying slope was deemed the best in model prediction accuracy.

Table S4

Model comparison values for the likelihood to change response model

Model	Elpd LOO (SE)	P LOO (SE)	LOO IC (SE)	Elpd diff. (SE)
Null model	-1529.1 (18.3)	75.6 (1.3)	3058.2 (36.6)	-444.5 (27.2)
One predictor	-1117.5 (28.2)	65.6 (2.2)	2235.1 (56.5)	-32.9 (8.2)
Additive model	-1111.3 (28.3)	53.1 (1.7)	2222.6 (56.6)	-26.2 (7.4)
Interaction model	-1092.4 (28.3)	56 (1.9)	2184.9 (56.5)	-7.8 (4.4)
Varying slope	-1084.6 (28.7)	67.1 (2.6)	2169.2 (57.4)	0.0 (0.0)

Note. Elpd = Expected log predictive density, SE = Standard error, P LOO = estimated effective number of parameters, LOO IC = Leave-one-out information criteria. One predictor refers to the model with only previous feedback added as a predictor.

For the sensitivity check, the model (with the varying slope for previous feedback and interaction term) was fitted with uninformed priors (default) to check if the effects were prior-driven or data-driven. Even with uninformed priors, the model replicated the same pattern (Table S5). All groups were more likely to change their response after a loss compared to a win, but this differs across syndromes. Participants with WS were more likely to repeat the same choice after a win than TD participants, whereas those with DS were less likely to do so in comparison to TD. The effects are stable and suggest that the results are data-driven rather than prior-dependent.

Table S5

Sensitivity check. Model fitted but with default priors. The intercept represents TD at negative feedback on the previous trial

Fixed effects	Estimate [95 % CI]	SD	OR [95 % CI]
Intercept (TD + Loss)	**1.43 [1.12, 1.78]**	0.17	**4.19 [3.06, 5.92]**
Previous feedback (Win)	**-3.43 [-3.88, -3.02]**	0.22	**0.03 [0.02, 0.05]**
DS	0.22 [-0.42, 0.87]	0.33	1.24 [0.66, 2.39]
FXS	0.36 [-0.32, 1.07]	0.36	1.44 [0.72, 2.93]
WS	**1.00 [0.33, 1.73]**	0.35	**2.72 [1.40, 5.61]**

Previous feedback* DS	**1.93 [1.04, 2.81]**	0.45	**6.87 [2.82, 16.59]**
Previous feedback* FXS	0.66 [-0.24, 1.55]	0.46	1.94 [0.78, 4.71]
Previous feedback *WS	**-0.90 [-1.74, -0.08]**	0.43	**0.41 [0.18, 0.92]**
**Random effects**			
SD (Intercept)	0.40 [0.03, 0.82]	0.22	
SD (Previous feedback)	0.83 [0.46, 1.22]	0.19	
Cor (Intercept, Previous feedback)	-0.20 [-0.82, 0.86]	0.45	

Note. Estimates are presented in logit values and are posterior means. TD = Typically Developed, DS = Down Syndrome, FXS = Fragile X Syndrome, WS = Williams Syndrome, CI = Credible interval, OR = Odds Ratio, SD = Standard Deviation. Bold indicates that 0 is not included in the 95% credible interval. Random effects represent variation across participants (participant level).

Pupil response and feedback valence

Six separate models were fitted. One with only feedback as a predictor, one with an interaction term (feedback * syndrome), one with block as an added predictor, one with the interaction term block * syndrome, one with trial added as a covariate (to the interaction term model), and one with varying slopes for feedback. Model comparisons were then carried out using the loo-package in R [4]. All Pareto *k* estimates were deemed good ($k < 0.7$). As can be seen (Table S6), the similarities between the models were high, but the model with the varying slope was deemed the best in model prediction accuracy.

Table S6

Model comparison values for pupil response

Model	Elpd LOO (SE)	P LOO (SE)	LOO IC (SE)	Elpd diff. (SE)
One predictor	-2252.3 (23.2)	3.7 (0.1)	4505.6 (46.4)	-6.2 (4.9)
Interaction model	-2252.2 (23.3)	9.2 (0.4)	4505.4 (46.5)	-6.1 (3.8)
Block added	-2246.3 (23.2)	10.3 (0.4)	4492.6 (46.5)	-0.1 (0.8)
Interaction syndrome*Block	-2248.2 (23.3)	13.3 (0.6)	4496.4 (46.7)	-2.0 (1.5)
Trial added	-2246.2 (23.3)	10.2 (0.4)	4492.3 (46.5)	0.0 (0.0)

Varying slope	-2247.2 (23.3)	11.7 (0.4)	4494.3 (46.5)	-1.0 (0.1)

Note. Elpd = Expected log predictive density, SE = Standard error, P LOO = estimated effective number of parameters, LOO IC = Leave-one-out information criteria. One predictor refers to the model with only feedback added as a predictor.

For the sensitivity check, the model (with the varying slope for feedback) was fitted with uninformed priors (default) to check if the effects were prior-driven or data-driven. Even with uninformed priors, the model replicated the same pattern (Table S7). The loss feedback elicited larger pupil responses in TD, but the difference between feedback types (in pupil response) was negligible in WS. The effects are stable and suggest that the results are data-driven rather than prior-dependent.

Table S7

Sensitivity check. Regression coefficients from the model using default priors. The intercept represents TD at loss feedback

**Fixed effects**	**Estimate [95% CI]**	**SD**
Intercept	**0.46 [0.31, 0.60]**	0.07
Feedback (Win)	**-0.38 [-0.52, -0.24]**	0.07
DS	-0.26 [-0.56, 0.05]	0.15
FXS	-0.27 [-0.61, 0.06]	0.17
WS	**-0.28 [-0.50, -0.06]**	0.11
Trial	**-0.01 [-0.02, -0.01]**	0.00
Feedback*DS	0.33 [-0.04, 0.71]	0.19
Feedback*FXS	0.33 [-0.09, 0.74]	0.21
Feedback*WS	**0.37 [0.11, 0.62]**	0.13
Sigma	0.96 [0.93, 0.99]	0.02
**Random effects**		
SD (Participant)	0.03 [0.0, 0.11]	0.03
SD (Feedback)	0.04 [0.00, 0.15]	0.04
Cor (Intercept, Slope)	-0.32 [-0.99, 0.89]	0.58

Note. Estimates are presented in logit values and are posterior means. TD = Typically Developed, DS = Down Syndrome, FXS = Fragile X Syndrome, WS = Williams Syndrome,

CI = Credible interval, SD = Standard Deviation. Bold indicates that 0 is not included in the 95% credible interval. Random effects represent variation across participants (participant level).

Gaze allocation and feedback valence

Four separate models were fitted. One with only previous feedback as a predictor, one with an interaction term (previous feedback * syndrome), one with trial added as a covariate (to the interaction term model), and one with varying slopes for feedback. Model comparisons were then carried out using the loo-package in R [4]. All Pareto *k* estimates were deemed good ($k < 0.7$). As can be seen (Table S8), the similarities between the models were high, but the model with the varying slope was deemed the best in model prediction accuracy.

Table S8

Model comparison values for gaze allocation

**Model**	**Elpd LOO (SE)**	**P LOO (SE)**	**LOO IC (SE)**	**Elpd diff. (SE)**
One predictor	12983.8 (342.0)	64.9 (2.6)	-25967.5 (683.9)	-20.7 (7.2)
Interaction model	12980.4 (341.9)	68.5 (2.7)	-25960.7 (683.7)	-24.1 (7.2)
Trial added	12987.2 (341.7)	69.3 (2.8)	-25974.3 (683.4)	-17.3 (6.5)
Varying slope (previous feedback)	13004.5 (341.8)	82.3 (3.7)	-26009.0 (683.6)	0.0 (0.0)

Note. Elpd = Expected log predictive density, SE = Standard error, P LOO = estimated effective number of parameters, LOO IC = Leave-one-out information criteria. One predictor refers to the model with only previous feedback added as a predictor.

For the sensitivity check, the model (with the varying slope for previous feedback) was fitted with uninformed priors (default) to check if the effects were prior-driven or data-driven. Even with uninformed priors, the model replicated the same pattern (Table S9). Participants overall looked longer at the option that was associated with a win compared to a loss ($b = 0.39$, 95% CI [0.23, 0.55]), and as the task progressed, participants looked less at the previously chosen option ($b = -0.02$, 95% CI [-0.02, -0.01]). No group differences or interaction effects emerged.

Table S9

Sensitivity check. Regression coefficients for gaze allocation using default priors

Parameter	Estimate [95% CI]	SD	OR [95% CI]
Intercept (TD + Loss)	**-1.13 [-1.31, -0.94]**	**0.09**	**0.32 [0.27, 0.39]**
Previous Feedback (Win)	0.42 [0.22, 0.63]	0.10	1.52 [1.25, 1.87]
DS	0.01 [-0.30, 0.32]	0.16	1.01 [0.74, 1.38]
FXS	-0.26 [-0.66, 0.14]	0.21	0.77 [0.52, 1.15]
WS	-0.12 [-0.39, 0.16]	0.14	0.89 [0.68, 1.17]
Trial	-0.02 [-0.02, -0.01]	0.00	0.98 [0.98, 0.99]
Previous Feedback (Win) * DS	0.02 [-0.45, 0.50]	0.24	1.02 [0.64, 1.39]
Previous Feedback (Win) * FXS	0.06 [-0.49, 0.62]	0.28	1.06 [0.62, 1.85]
Previous Feedback (Win) * WS	0.05 [-0.33, 0.42]	0.19	1.05 [0.72, 1.53]
Phi	0.38 [0.36, 0.40]	0.01	
**Random effect**			
SD (Participant)	0.15 [0.02, 0.29]	0.07	
SD (Previous feedback)	0.48 [0.31, 0.64]	0.08	
Cor (Intercept, Slope)	0.61 [-0.22, 0.99]	0.33	

Note. Estimates are presented in logit values and are posterior means. TD = Typically Developed, DS = Down Syndrome, FXS = Fragile X Syndrome, WS = Williams Syndrome, CI = Credible interval, OR = Odds ratio, SD = Standard deviation. Bold indicates that 0 is not included in the 95% credible interval. Random effects represent variation across participants (participant level).

Does stimulus type have an impact on the different outcomes?

To analyze whether stimulus type (face or figure) affected the outcomes, the chosen models were fitted with stimulus type added as a predictor, as well as its interaction with syndrome (stimulus type * syndrome), due to the special interest WS have for faces [5]. Stimulus type did not have an impact on either the likelihood of changing response or pupil response (Tables S9-10), but it did have an impact on gaze allocation (Table S11). Participants had a higher fixation proportion on the previous option in the face stimulus pairs than in the figure stimulus pairs. There was no difference between the groups (see Figure S5).

Table S10

Fixed and random effects for the models for feedback learning, with stimulus type added as a predictor

**Fixed effects**	**Estimate [95% CI]**	**SD**
Intercept (TD + Loss + Figure)	**1.33 [1.03, 1.63]**	0.15
Previous Feedback (Win)	**-3.05 [-3.39, -2.72]**	0.17
DS	0.39 [-0.15, 0.93]	0.27
FXS	0.34 [-0.28, 0.96]	0.31
WS	**0.82 [0.29, 1.37]**	0.28
Stimulus type (Face)	-0.05 [-0.40, 0.30]	0.18
Previous Feedback (Win) * DS	**1.50 [0.77, 2.20]**	0.36
Previous Feedback (Win) * FXS	0.37 [-0.36, 1.09]	0.37
Previous Feedback (Win) * WS	**-0.85 [-1.51, -0.22]**	0.33
Stimulus Type (Face) * DS	-0.36 [-1.22, 0.50]	0.44
Stimulus Type (Face) * FXS	0.07 [-0.73, 0.88]	0.41
Stimulus Type (Face) * WS	-0.16 [-0.80, 0.50]	0.33
**Random effect**		
Intercept (Participant)	0.27 [0.02, 0.62]	0.17
Slope (Previous feedback)	0.72 [0.40, 1.03]	0.16
Cor (Intercept, Slope)	0.02 [-0.68, 0.79]	0.40

Note. Estimates are presented in logit values. TD = Typically Developed, DS = Down Syndrome, FXS = Fragile X Syndrome, WS = Williams Syndrome, CI = Credible Interval, SD = Standard Deviation. Bold indicates that 0 is not included in the 95% credible interval. Random effects represent variation across participants (participant level).

Table S11

Fixed and random effects for pupil response, with stimulus type added as a predictor

**Parameter**	**Estimate [95% CI]**	**SD**
Intercept (TD + Loss + Figure)	**0.44 [0.28, 0.59]**	0.08
Feedback (Win)	**-0.37 [-0.50, -0.23]**	0.07
DS	-0.22 [-0.56, 0.12]	0.17
FXS	-0.29 [-0.68, 0.09]	0.20

WS	-0.17 [-0.48, 0.19]	0.12
Trial	**-0.01 [-0.02, -0.01]**	0.00
Stimulus type (Face)	0.03 [-0.09, 0.15]	0.06
Feedback (Win) * DS	0.30 [-0.06, 0.64]	0.18
Feedback (Win) * FXS	0.27 [-0.11, 0.66]	0.20
Feedback (Win) * WS	**0.35 [0.09, 0.60]**	0.13
Stimulus Type (Face) * DS	-0.02 [-0.37, 0.33]	0.18
Stimulus Type (Face) * FXS	0.10 [-0.31, 0.50]	0.21
Stimulus Type (Face) * WS	-0.17 [-0.41, 0.05]	0.12
Sigma	0.96 [0.93, 0.99]	0.02
**Random effect**		
Intercept (Participant)	0.02[0.00, 0.07]	0.02
Slope (Feedback)	0.03 [0.00, 0.10]	0.03
Cor (Intercept, Slope)	-0.16 [-0.90, 0.74]	0.46

Note. Estimates are presented in z-transformed values for pupil response. TD = Typically Developed, DS = Down Syndrome, FXS = Fragile X Syndrome, WS = Williams Syndrome, CI = Credible Interval. Bold indicates that 0 is not included in the 95% credible interval. Random effects represent variation across participants (participant level).

Table S12

Fixed and random effects for fixation proportion, with stimulus type added as a predictor

**Parameter**	**Estimate [95% CI]**	**SD**
Intercept (TD + Loss + Figure)	**-1.24 [-1.44, -1.04]**	0.10
Previous Feedback (Win)	**0.43 [0.23, 0.63]**	0.10
DS	-0.00 [-0.39, 0.36]	0.19
FXS	-0.39 [-0.91, 0.12]	0.26
WS	0.01 [-0.31, 0.32]	0.16
Trial	**-0.02 [-0.02, -0.01]**	0.00
Stimulus type (Face)	**0.24 [0.06, 0.42]**	0.09
Prev. Feedback (Win) * DS	0.05 [-0.41, 0.51]	0.23
Prev. Feedback (Win) * FXS	0.06 [-0.47, 0.59]	0.27
Prev. Feedback (Win) * WS	0.04 [-0.33, 0.41]	0.19

Stimulus Type (Face) * DS	0.08 [-0.43, 0.58]	0.26
Stimulus Type (Face) * FXS	0.18 [-0.39, 0.75]	0.29
Stimulus Type (Face) * WS	-0.24 [-0.57, 0.09]	0.17
Phi	0.38 [0.36, 0.40]	0.01
**Random effect**		
Intercept (Participant)	0.14 [0.01, 0.29]	0.07
Slope (Previous feedback)	0.48 [0.32, 0.63]	0.08
Cor (Intercept, Slope)	0.41 [-0.30, 0.91]	0.32

Note. Estimates are presented in logit values. TD = Typically Developed, DS = Down Syndrome, FXS = Fragile X Syndrome, WS = Williams Syndrome, CI = Credible Interval. Bold indicates that 0 is not included in the 95% credible interval. Random effects represent variation across participants (participant level).

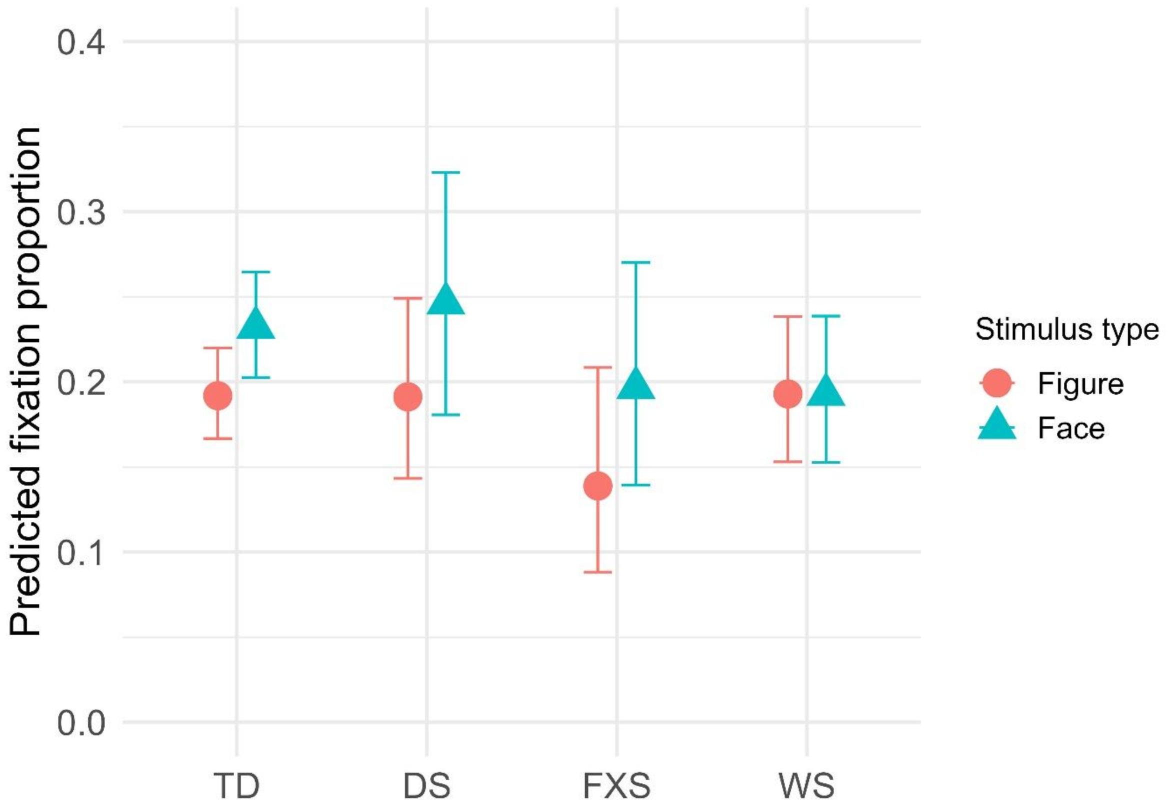

Figure S5. Conditional effects for the interaction effect between stimulus type and syndrome. TD = Typically Developed, DS = Down Syndrome, FXS = Fragile X Syndrome, WS = Williams Syndrome.

Does gaze allocation predict feedback learning behaviors?

To examine whether gaze allocation predicts response changes, we fitted a GLMM for each syndrome with an interaction term (previous feedback * fixation proportion on the previous choice) on trial level, with varying intercepts for participants and varying slopes for previous feedback. As shown in Table S13, there was strong evidence that fixation proportion affected response behavior in TD (b = -0.61, 95% CI = [-1.08, -0.15], BF = 21.40, pd = 1) and DS participants (b = -0.59, 95% CI = [-1.10, -0.06], BF = 9.90, pd = 0.98), suggesting that a higher proportion of fixation duration on the previously chosen option made participants *less likely to change response*. FXS and WS individuals were less likely to change their response the more they looked at the previously chosen option, although evidence for this effect was less robust. There was also no evidence for an interaction effect (Table S13) for any of the syndrome groups. For TD, there was also an interaction effect indicating that after positive feedback (win), the higher the fixation proportion on the previously chosen (win) option, the less likely they were to change response (b = -0.74, 95% CI = [-1.41, -0.07], BF = 7.27, pd = 0.98). All groups showed very strong and robust evidence for a higher likelihood of changing response after negative feedback (loss), except for DS participants. They were less likely to change response after a win but with less robust evidence (b = -0.66, 95% CI = [-1.34, 0.03], BF = 3.21, pd = 0.97).

Table S13

Likelihood of changing response on the previous trial predicted by gaze allocation per syndrome

**Fixed effects**	**Estimate [95 % CI]**	**SD**	**OR [95 % CI]**	**BF**	**pd**	**p-value**
TD						
Intercept	**1.21 [0.95, 1.49]**	0.14	3.36 [2.59, 4.44]	**>999**	1	<.001
Prev. Feedback (Win)	**-2.70 [-3.03, -2.37]**	0.17	0.06 [0.05, 0.36]	**>999**	1	<.001
Fixation proportion	-0.61 [-1.08, -0.15]	0.24	0.54 [0.34, 0.86]	**21.40**	0.99	.045
Fixation proportion * Prev. Feedback (Win)	-0.74 [-1.41, -0.07]	0.34	0.48 [0.24, 0.94]	**7.27**	0.98	.099
**Random effects**						
SD (Intercept)	0.13 [0.00, 0.40]	0.11				
SD (Slope)	0.21 [0.01, 0.61]	0.17				

Cor (Intercept, slope)	-0.22 [- 0.98, 0.91]	0.58				
DS						
Intercept	**1.09 [0.56, 1.68]**	0.28	2.99 [1.75, 5.34]	**367.45**	1	<.001
Prev. Feedback (Win)	**-0.66 [-1.34, 0.03]**	0.35	0.51 [0.26, 1.03]	**3.12**	0.97	.157
Fixation proportion	**-0.59 [-1.10, -0.06]**	0.27	0.56 [0.33, 0.94]	**9.90**	0.99	.096
Fixation proportion * Prev. Feedback (Win)	-0.46 [-1.27, 0.34]	0.41	0.63 [0.28, 1.41]	**1.54**	0.87	.539
**Random effects**						
SD (Intercept)	0.43 [0.02, 1.63]	0.32				
SD (Slope)	1.18 [0.29, 2.25]	0.49				
Cor (Intercept, slope)	-0.07 [-0.92, 0.92]	0.53				
FXS						
Intercept	**1.20 [0.36, 2.07]**	0.43	**3.34 [1.43, 7.90]**	**14.56**	1	.026
Prev. Feedback (Win)	**-1.36 [-2.06, -0.60]**	0.37	**0.26 [0.13, 0.55]**	**144.49**	1	.003
Fixation proportion	-0.24 [-0.78, 0.30]	0.28	0.79 [0.46, 1.35]	1.33	0.80	.700
Fixation proportion * Prev. Feedback (Win)	-0.54[-1.36, 0.28]	0.42	0.58 [0.26, 1.32]	2.01	0.90	.438
**Random effects**						
SD (Intercept)	1.03 [0.11, 2.11]	0.49				
SD (Slope)	0.62 [0.02, 1.83]	0.50				
Cor (Intercept, slope)	0.13 [-0.89, 0.96]	0.54				
WS						
Intercept	**1.56 [1.07, 2.06]**	0.25	**4.76 [2.92, 7.82]**	**>999**	1	<.001
Prev. Feedback (Win)	**-2.78 [-3.35, -2.17]**	0.30	**0.06 [0.03, 0.11]**	**>999**	1	<.001
Fixation proportion	-0.15 [-0.68, 0.37]	0.27	0.86 [0.51, 1.45]	1.05	0.71	.860
Fixation proportion * Prev. Feedback (Win)	-0.30 [-1.03, 0.41]	0.37	0.74 [0.36, 1.51]	1.02	0.79	.713
**Random effects**						
SD (Intercept)	0.43 [0.02, 0.99]	0.27				
SD (Slope)	0.64 [0.03, 1.52]	0.40				
Cor (Intercept, slope)	0.12 [-0.88, 0.96]	0.54				

Note. TD = Typically Developed, DS = Down Syndrome, FXS = Fragile X Syndrome, WS = Williams Syndrome, BF = Bayes factor (BF10), pd = Probability of direction, CI = Credible interval, SD = Standard deviation, OR = Odds ratio. The estimates are posterior means and

are presented in logit values together with 95% credible intervals. Bold indicates that 0 is not included in the 95% credible interval.

Does the proportion of looking time on the different AOIs differ between the groups?

WS and DS individuals have more difficulties orienting visual attention to the periphery compared to TD individuals [6,7]. An exploratory analysis was conducted to examine whether the syndrome groups had a higher proportion of fixation on the center Area of interest (AOI). We fitted a GLMM with a Beta distribution and a logit link on trial level, using syndrome as a predictor and including a random intercept for participant. Since prior research [7] has shown moderate to large effects regarding individuals with a syndrome having more difficulty shifting attention, the prior for the intercept was normal(0, 1) and the same for the predictor. The prior for phi was exponential(2) and exponential(1) for the random effects. Results showed that TD individuals exhibited above-chance proportions of looking at the center AOI (b = 0.33, 95% CI [0.13, 0.52], BF = 15.78, pd = 1). In contrast, DS individuals look less at the center AOI compared to TD individuals (b = -0.61, 95% CI [-1.06, -0.16], BF = 7.18, pd = 1). FXS and WS individuals did not differ in their looking pattern from TD, suggesting they also looked above chance proportions at the center AOI (FXS: b = 0.03, 95% CI [-0.45, 0.51], BF = 0.21; WS: b = 0.08, 95% CI [-0.25, 0.43], BF = 0.17).

References

1. Bochud-Fragnière, E. *et al.* Why do individuals with Williams syndrome or Down syndrome fail the Weather Prediction Task? *Dev. Psychobiol.* **66**, e22503 (2024).
2. Guath, M., Willfors, C., Björlin Avdic, H., Nordgren, A. & Kleberg, J. L. Pupillary response in reward processing in adults with major depressive disorder in remission. *J. Int. Neuropsychol. Soc. JINS* **29**, 306–315 (2023).
3. Balcazar, J. & Orr, J. M. The role of uncertain reward in voluntary task-switching as revealed by pupillometry and gaze. *Behav. Brain Res.* **480**, 115403 (2025).
4. Vehtari, A., Gelman, A. & Gabry, J. Practical Bayesian model evaluation using leave-one-out cross-validation and WAIC. *Stat. Comput.* **27**, 1413–1432 (2017).
5. Riby, D. M. *et al.* Attention to Faces in Williams Syndrome. *J. Autism Dev. Disord.* **41**, 1228–1239 (2011).

6. D'Souza, D., D'Souza, H., Jones, E. J. H. & Karmiloff-Smith, A. Attentional abilities constrain language development: A cross-syndrome infant/toddler study. *Dev. Sci.* **23**, e12961 (2020).
7. Hallman, A. *et al.* Challenges with shifting, regardless of disengagement: attention mechanisms and eye movements in Williams syndrome. *J. Neurodev. Disord.* **17**, 48 (2025).
